# Low-Energy Electron Potentiometry: Contactless Imaging of Charge Transport on the Nanoscale

**DOI:** 10.1038/srep13604

**Published:** 2015-09-04

**Authors:** J. Kautz, J. Jobst, C. Sorger, R. M. Tromp, H. B. Weber, S. J. van der Molen

**Affiliations:** 1 Leiden University, Huygens-Kamerlingh Onnes Laboratory, P.O. Box 9504, NL-2300 RA Leiden, Netherlands; 2 Lehrstuhl für Angewandte Physik, Universität Erlangen-Nürnberg, 91058 Erlangen, Germany; 3 IBM T.J. Watson Research Center, 1101 Kitchawan Road, P.O. Box 218, Yorktown Heights, New York 10598, US

## Abstract

Charge transport measurements form an essential tool in condensed matter physics. The usual approach is to contact a sample by two or four probes, measure the resistance and derive the resistivity, assuming homogeneity within the sample. A more thorough understanding, however, requires knowledge of local resistivity variations. Spatially resolved information is particularly important when studying novel materials like topological insulators, where the current is localized at the edges, or quasi-two-dimensional (2D) systems, where small-scale variations can determine global properties. Here, we demonstrate a new method to determine spatially-resolved voltage maps of current-carrying samples. This technique is based on low-energy electron microscopy (LEEM) and is therefore quick and non-invasive. It makes use of resonance-induced contrast, which strongly depends on the local potential. We demonstrate our method using single to triple layer graphene. However, it is straightforwardly extendable to other quasi-2D systems, most prominently to the upcoming class of layered van der Waals materials.

The past years have seen a tremendous increase in available quasi-2D materials, extending from graphene^1^ to van der Waals heterostructures^2^ and topological insulators^3^,^4^,^5^. Not only have remarkable physical phenomena such as Dirac-Weyl physics^6^,^7^ and Klein tunneling^8^ been observed, these materials also offer great opportunities for applications in electronic devices^1^,^3^. To maximize their potential, precise knowledge of the local electron transport properties is essential. In topological insulators for instance, the conductance is completely governed by edge states, while the bulk remains insulating^5^. In graphene, on the other hand, charge transport can be dominated by electron and hole puddles created by the intimate contact to a substrate^9^. Furthermore, small-scale variations like step edges, grain boundaries and atomic defects can strongly affect global properties. To elucidate such local conductance properties, several groups have performed ground-breaking experiments using scanning probe techniques such as Kelvin probe microscopy^10^, (four-probe) scanning tunneling microscopy^11^ and scanning squid microscopy^12^. The scanning nature of these techniques, however, inherently leads to long acquisition times and a limited field of view. Here, we introduce a novel tool, coined low-energy electron potentiometry (LEEP), which allows for rapid imaging of potential landscapes, with both high resolution and a field of view of up to 10 μm. From the known performance of the microscope^13^, we estimate <5 nm spatial resolution and ~10 meV energy resolution to be achievable with LEEP. The technique does not require a local, possibly invasive probe. Moreover, the entire field of view is imaged at once, reducing the acquisition time for a potential map below one minute. Hence, potentiometry studies of dynamic processes come within reach. Interestingly, LEEP experiments can be combined with Low-Energy Electron Microscopy (LEEM)^14^ and Photo Electron Emission Microscopy and Spectroscopy (PEEM) measurements on the same sample in the same microscope. This combination forms an extremely powerful set of complementary tools, LEEM allowing one to determine the structure and morphology of 2D materials, PEEM giving access to the electronic band structure, and LEEP providing insight in charge transport on the nanoscale.

## Method

**Figure 1:**
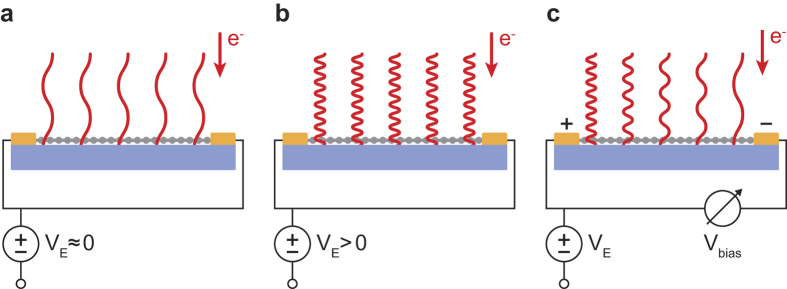
The local landing energy of incident electron waves (indicated by red lines) depends on the local electric potential of the graphene (gray) on the silicon carbide substrate (blue). (**a**) For an overall sample potential *V*_*E*_ ≈ 0, the electrons barely reach the sample, i.e. their landing energy is almost zero and their wavelength is long. (**b**) By applying a voltage *V*_*E*_ to the whole sample the landing energy can be increased, thereby decreasing the electron wavelength. (**c**) An in-plane bias voltage *V*_bias_ applied over the sample changes the local sample potential. Hence, the landing energy and the electron wavelength become position-dependent. Here, the situation for *V*_bias_ < 0 is shown, i.e. the right electrode is at a more negative potential, which resembles the experimental situation presented.

The basic idea behind LEEP is to determine the local potential at each position on a sample from LEEM images, i.e., from the intensity of specularly reflected low-energy electrons (0–100 eV) that are projected onto a pixelated detector^14^,^15^,^16^,^17^. The landing energy of the electrons at the surface can be tuned by varying the overall sample potential *V*_*E*_ (Fig. 1a,b). Importantly, for each material the electron reflection probability depends strongly on the electron landing energy or, equivalently, on the electron wavelength λ as given by the de Broglie relation. In fact, a measurement of the signal intensity *I* versus *V*_*E*_, yielding a so-called IV-curve *I*(*V*_*E*_), is considered a fingerprint of a specific surface structure^18^,^19^. In a conductance experiment, an in-plane bias voltage *V*_*bias*_ is applied over a sample. This causes the local electron landing energy (wavelength) to become position-dependent, as depicted in Fig. 1c. Consequently, for each point on the sample, the local IV-curve is shifted in energy^20^. By quantifying this shift, one can determine a full surface potential map. This is the essence of the potentiometry method introduced here.

In principle, any clean material has its characteristic IV-curve and can thus be studied by potentiometry. Still, LEEP is expected to work best if the intensity exhibits a strong and well-defined energy dependence. The layered structure of van der Waals heterostructures, for example, has interesting consequences for LEEM IV-curves. The properties of these stacks of 2D systems like hexagonal boron nitride, graphene or transition metal dichalcogenides, held together by van der Waals forces, are tunable by choosing a specific stacking^2^. In these materials, there typically exist unoccupied states, localized between adjacent layers^18^. The electronic coupling between these interlayer states causes a splitting of their energy levels, resulting in *n* non-degenerate states for materials with *n* + *1* layers. When the electron landing energy in a LEEM experiment is equal to the energy of one of these states, resonant coupling between the electron wave and the interlayer state suppresses electron reflection. This yields minima in the IV-curve, with the number of such minima corresponding to the number of interlayer states *n*. For graphene on silicon carbide (SiC), for example, the number of minima corresponds to the number of conducting graphene layers, because the bottommost carbon layer is a buffer layer that is insulating, but does contribute to the formation of interlayer states^21^. Thus, LEEM IV-curves form a powerful tool to characterize novel van der Waals systems. Moreover they can be used as a basis for potentiometry.

**Figure 2:**
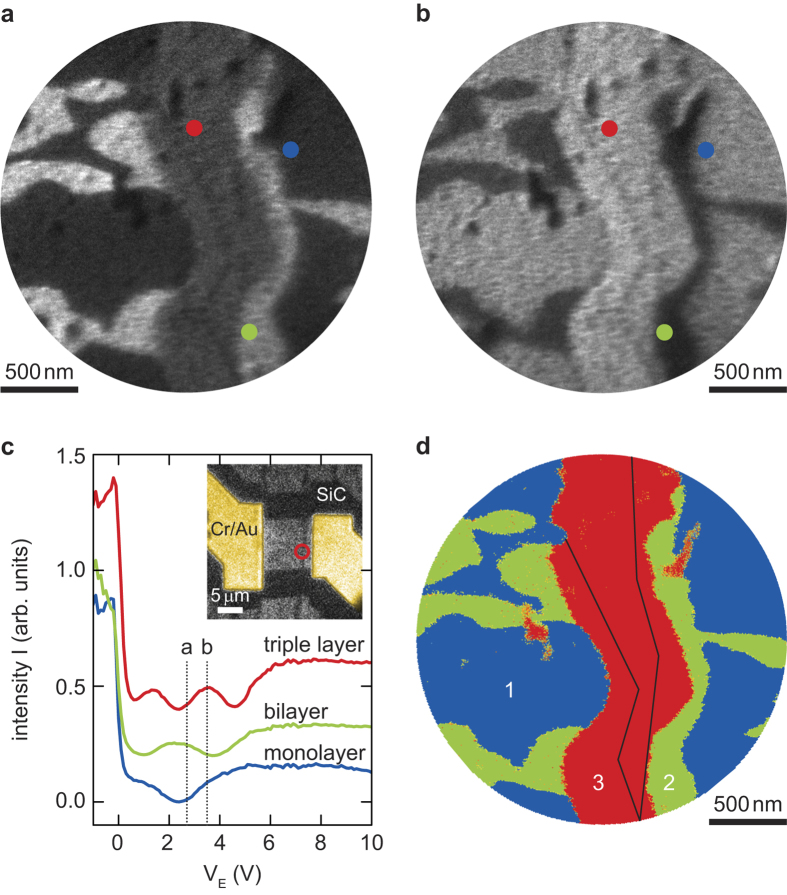
Resonant interaction of electron waves with graphene results in a strongly energy-dependent LEEM contrast. (**a**) Bilayer areas appear bright, while monolayer and triple layer regions appear dark in LEEM images (bright-field) taken at an electron energy of *V*_*E*_ = 2.7 V. (**b**) For LEEM images taken at *V*_*E*_ = 3.5 V the same features as in (**a)** are visible but the contrast is inverted. (**c**) The IV-curves taken at positions indicated in (**a**,**b)** exhibit minima due to resonant absorption of electrons by unoccupied states between graphene layers. For graphene on SiC, the number of minima corresponds to the number of conducting graphene layers. The curves are offset in intensity for clarity. The dotted lines indicate the electron energy in (**a**,**b**). The inset shows a photoemission electron microscopy image of the device presented. SiC step edges are clearly visible as dark lines. The red circle indicates the field of view for the presented LEEM images. (**d)** A map of graphene thickness can be obtained by studying IV-curves pixel by pixel. Enhanced graphene growth is observed near SiC step edges (black lines), which are also visible in (**a**,**b**).

To explore such ‘resonant’ IV-curves, we use multilayer graphene as a model system for van der Waals materials. Particularly, we choose graphene grown on SiC, because it is clean and homogeneous over large areas and has a well-defined step direction^22^. Electrical contact to a graphene device, patterned perpendicular to the SiC steps, is made via lithographically defined gold electrodes. Subsequently, remaining resist is thoroughly removed (see Supplementary Methods: Sample Preparation). Figure 2a,b, show LEEM images of the same area of the graphene device (circle in inset of Fig. 2c) acquired at two different electron landing energies with no in-plane bias applied. While the same features can be distinguished in both images, the contrast differs considerably. This can be understood by looking at the IV-curves presented in Fig. 2c. These curves, taken at three different spots of the sample, exhibit 1, 2 and 3 minima respectively, which, as discussed above, corresponds to monolayer, bilayer and triple layer of electronically well-defined graphene. This allows us to tune the contrast of LEEM images by changing the electron landing energy. For *V*_*E*_ = 2.7 V (Fig. 2a), the bilayer IV-curve exhibits a maximum (appears bright), as the triple layer curve shows a minimum (appears dark), while the energy used in Fig. 2b (*V*_*E*_ = 3.5 V) causes contrast inversion. We have taken IV-curves at every point of this field of view by recording LEEM images while sweeping *V*_*E*_ (see Supplementary Video 1). From the number of minima in these IV-curves, we deduced the local graphene thickness experimentally, resulting in the spatial map in Fig. 2d. It shows enhanced graphene growth around the SiC step edges visible in Fig. 2a,b (indicated by black lines in d). The ability to accurately distinguish step edges as well as local thickness variations of layered materials is one of the exciting features of LEEM/LEEP.

**Figure 3:**
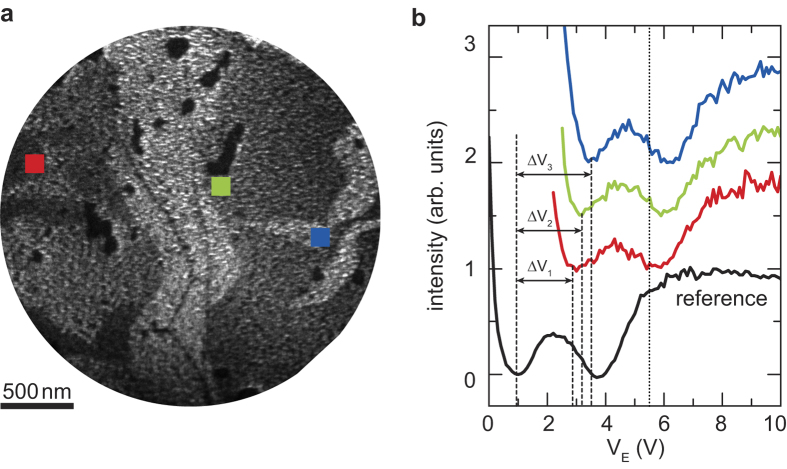
The local IV-curves are shifted in energy due to local potential differences. (**a**) LEEM image taken at *V*_*E*_ = 5.4 V with a sample bias of *V*_bias_ = −3 V. Due to the bias the landing energy becomes position dependent (see Fig. 1c). Hence, bilayer areas on the left (ground side) appear dark, while they are bright at the right (bias side). (**b**) IV-curves taken at bilayer areas from single pixels in the areas indicated by squares in (**a**). They show the two characteristic minima but are shifted with respect to the reference curve obtained from the unbiased case in Fig. 2c. The shifts Δ*V* are a direct measure for the local potential. The curves are offset in intensity for clarity. The dotted line indicates the electron energy in (**a)**.

We next use the rich structure of the graphene IV-curves to precisely measure local potential values. In such a LEEP experiment, we apply an in-plane bias voltage *V*_*bias*_ = −3 V over the sample as sketched in Fig. 1c. Next, we acquire LEEM images while sweeping *V*_*E*_ (see Supplementary Video 2). Figure 3a shows a snapshot taken at *V*_*E*_ = 5.4 V. The differences between the unbiased case (Fig. 2a,b) and the biased case (Fig. 3a) are immediately visible. While, for example, bilayer areas show the same intensity for the entire field of view in the unbiased situation, they appear darker on the left than on the right in the biased case. The latter is a direct result of the landing energy being larger at the left side than at the right side of the image, which causes an energy shift Δ*V* of the local IV-curves and thus a difference in the local image intensity. To quantify this effect, we have measured an IV-curve for every pixel in the image. Figure 3b shows three of these IV-curves, taken within the bilayer areas indicated in Fig. 3a. They all have a similar shape, featuring two minima, but are shifted in energy with respect to each other as well as to a reference IV-curve taken for the unbiased case. This shift Δ*V* is a direct measure for the local potential *V(x, y)*. Note that the distinct shape of the IV-curves due to the resonant coupling to interlayer graphene states allows us to deduce this shift particularly precisely and thus enhances the resolution of the LEEP technique. Similar minima are expected for many layered quasi-2D crystals. In fact, most materials show clear structure in LEEM-IV^19^. The method to compute the shift using features in the IV-curve can therefore easily be extended to other quasi-2D systems. In particular, utilizing such features yields better results than taking the steep drop of the mirror mode transition as a measure for the local electrostatic potential (see Supplementary Note).

## Results and Discussion

**Figure 4:**
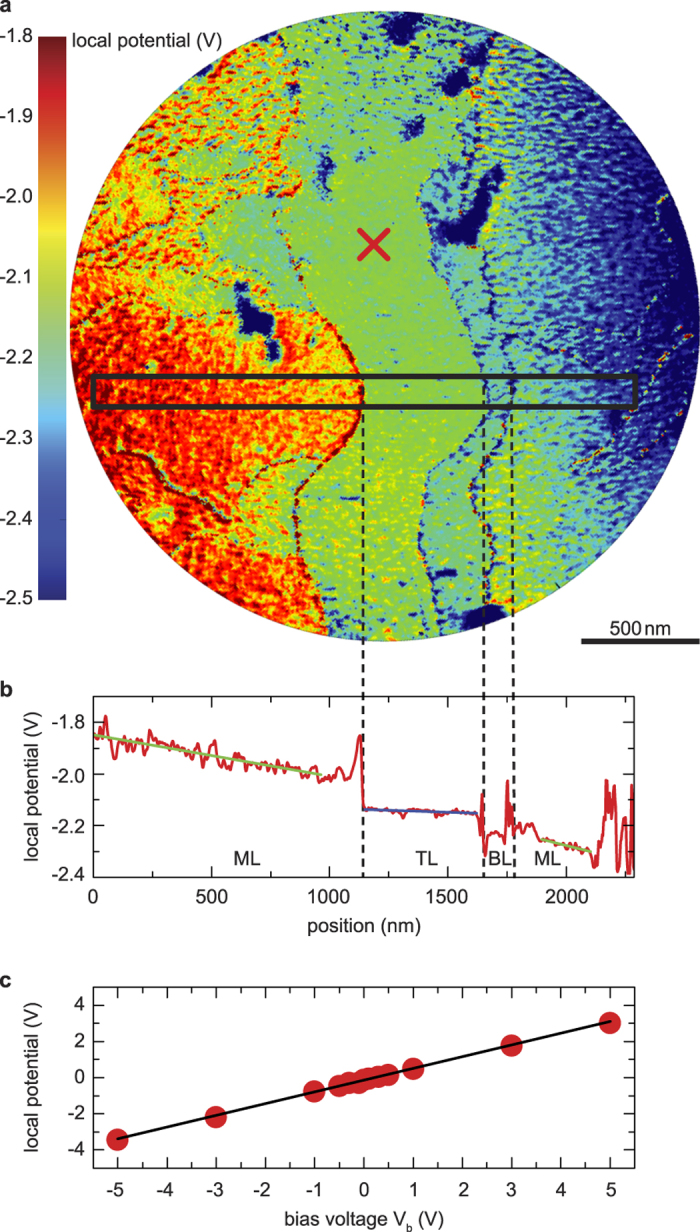
The local electric potential of a biased graphene sample can be mapped out using LEEP. (**a**) potential map of the sample at *V*_bias_ = −3 V bias is obtained by pixelwise calculating the shift Δ*V* of IV-curves with respect to a zero bias reference. (**b**) A linescan over the potential map in (**a)** shows the voltage drop over the sample. The linear gradient in the monolayer area is smaller than that in the triple layer area, indicating a lower resistivity for the latter. At the interface between single and triple layer graphene, a sharp drop in the local potential is observed, while the macroscopic SiC step edges remain barely visible in the potential image. The spikes at the interface between areas with different thicknesses are artifacts, caused by image drift during image acquisition. (**c**) Potential at the position indicated by a cross in (**a)** as a function of applied bias voltage *V*_bias_. The linear relation confirms the metallic properties expected for graphene and shows that we probe the bias dependent, electric potential only. The deviations from this linear trend (~50 mV) form an upper limit for our absolute potential resolution.

A complete potential map of the sample can now be produced by comparing the IV-curve at every pixel with a reference IV-curve taken at zero bias (Technical details of the algorithm used can be found in the Supplementary Methods: Shift Determination). Figure 4a presents a map of the local potential *V(x, y)*, derived using the LEEP technique. The grainy structure of the image is mainly caused by residues of the resist used and is considered noise in the following. As expected for an Ohmic material like graphene, a voltage drop from left to right is apparent.

A potential profile along the indicated line (see Fig. 4b) show several remarkable features. First, the potential gradient and hence the resistivity within the triple layer is considerably lower than within the single layer. This is consistent with previous experimental reports and can be related to both the increased thickness and the protection of the bottom layers from doping from the ambient^23^. Second, we find no additional voltage drop at the macroscopic (5–10 nm high^22^) step edges of the SiC substrate below the triple layer graphene. Whereas these step edges are clearly resolved in LEEM images (cf. Fig. 3a) as thin dark lines within the triple layer area, they are barely visible in the potential map and the potential gradient in Fig. 4a,b. This indicates that no significant scattering occurs at these substrate steps that are covered with graphene in a carpet-like manner^24^. Remarkably, we do find a voltage drop of ~0.1 V at points where the graphene layer thickness changes. The latter is in agreement with scanning probe studies that relate this effect to a wave function mismatch between graphene of different layer number^11^,^25^,^26^.

One key factor in our experiment is LEEM’s ability to discriminate steps in the SiC substrate from a change in graphene layer number. This distinction is particularly challenging for other techniques, as graphene layer changes can coincide with SiC steps and are mainly found in the vicinity of macroscopic SiC step edges, where graphene growth is faster^22^. Consequently, standard conductivity experiments attributed the additional resistivity found to these macroscopic steps rather than to the graphene-induced steps^27^,^28^. Using LEEP, we find direct evidence that this conclusion is incorrect, demonstrating the added value of this local technique.

To quantify the energy resolution of LEEP, and as a consistency check, we acquired potential maps for different external bias voltages *V*_*bias*_. A linear relation between bias voltage and local potential is expected according to Ohm’s law. Figure 4c shows the measured local potential for the spot indicated in Fig. 4a as a function of *V*_*bias*_. Deviations from the apparent linear trend yield an estimate for the uncertainty in the absolute value of the local potential of ~50 mV. Interestingly, a higher resolution can be obtained for the relative potential for a single bias voltage. We estimate errors in relative potential of 25 mV or 7 mV for monolayer or triple layer areas, respectively, from the noise in the linescan in Fig. 4b. This error is mainly caused by residues of the organic resist used during sample fabrication. These residues also limit the lateral resolution of our technique. If this issue could be overcome, we expect a resolution of LEEP of <5 nm given the resolution limit of the microscope of 1.4 nm^29^. Consequently, LEEP provides a complementary tool to existing scanning tunneling potentiometry (STP) set-ups that allow for sub-nanometer spatial resolution and microvolt potential resolution^26^,^30^. STP, however, relies on a charged, rigid probe in the vicinity of the sample that can influence the electron paths^31^,^32^ and local potentials^33^,^34^,^35^ and therefore, can create artifacts. Moreover, the scanning nature of STP leads to long acquisition times of multiple days per potential map^30^, thus posing high demands on sample and tip stability. With LEEP, in contrast, measuring a potential map takes less than a minute. The sample is almost not disturbed by the measurement as the LEEP image is formed with a probing beam current density of 5 pA/μm^2^. In addition, LEEP offers a wide field of view of up to 10 μm with the option to zoom in, thus making it the ideal tool for typical device dimensions.

## Conclusions

In summary, we present a new method to analyze the laterally resolved conductivity of 2D systems. The LEEP technique introduced is based on the absorption of low-energy electrons resonant with unoccupied states in layered materials. It is fast and does not rely on an invasive, local probe. We demonstrate our technique by analyzing the conductance properties of few-layer graphene. We find an additional resistance contribution at points where the graphene layer thickness changes, while macroscopic steps in the SiC substrate do not perturb the current. We anticipate that LEEP is easily extended to other quasi-2D systems like van der Waals heterostructures and topological insulators. Moreover, given the recent developments in LEEM acquisition speed^36^, potentiometry of dynamic processes comes within range.

## Additional Information

**How to cite this article**: Kautz, J. *et al.* Low-Energy Electron Potentiometry: Contactless Imaging of Charge Transport on the Nanoscale. *Sci. Rep.* **5**, 13604; doi: 10.1038/srep13604 (2015).

## Supplementary Material

Supplementary Information

Supplementary Information

Supplementary Information
