# Supplementary material for: Low-Energy Electron Potentiometry: Contactless Imaging of Charge Transport on the Nanoscale
Source: Sci Rep. 2015 Sep 4;5:13604. doi: 10.1038/srep13604 (PMC4559764; doi:10.1038/srep13604)
Supplement: Supplementary Information [file srep13604-s1.pdf]

Supplementary Information for:

Low-Energy Electron Potentiometry: Contactless Imaging of Charge Transport on the Nanoscale

J. Kautz[1†], J. Jobst[1†], C. Sorger[2], R. M. Tromp[3,1], H. B. Weber[2] and S. J. van der Molen[1*]

[1]Leiden University, Huygens-Kamerlingh Onnes Laboratory, P.O. Box 9504, NL-2300 RA Leiden, Netherlands.

[2]Lehrstuhl für Angewandte Physik, Universität Erlangen-Nürnberg, 91058 Erlangen, Germany.

[3]IBM T.J. Watson Research Center, 1101 Kitchawan Road, P.O. Box 218, Yorktown Heights, New York 10598, US.

*Correspondence to: molen@physics.leidenuniv.nl

† These authors contributed equally.

This file includes:

Supplementary Methods
Supplementary Note
Supplementary Fig. 1
Supplementary Fig. 2
Captions for Supplementary Video 1 and 2

Other Supplementary Information for this manuscript includes the following:

Supplementary Video 1
Supplementary Video 2

Supplementary Methods

Sample Preparation

Commercial semi-insulating 4H SiC substrates are annealed in a SiC container at 1650°C. Subsequently, graphene is grown by thermal decomposition in argon atmosphere at 0.9bar pressure and at 1700°C following the procedure described by Emtsev et al.[21]. Devices are defined on the chip by electron beam lithography. We use 950k PMMA as resist for the patterning of graphene as well as for the fabrication of metallic contacts. For the former, areas where graphene is to be removed are illuminated and developed in MIBK/IPA (1:3). The remaining resist serves as etching mask during the oxygen plasma etch that removes graphene in the exposed areas. Subsequently, contacts are defined in a separate lithography step by thermal evaporation of chromium (5nm) and gold (20nm), followed by a resist lift-off in acetone. The graphene is partially etched away below the contacts to increase the adhesion of the contact material to the substrate without sacrificing the low contact resistance to graphene.

After processing, the samples are thoroughly cleaned in acetone and isopropanol and blown dry with nitrogen to minimize the contamination with resist residues.

Experimental Setup

Low energy electron microscopy (LEEM) is performed in the aberration-corrected LEEM facility, ESCHER, in Leiden, which is described in detail in an earlier publication[16]. Electrons are transported at a column energy of 15 keV, and decelerated across the 1.5 mm objective lens-sample gap, with the sample at a potential of -15 kV $+V_E$. Hence, the electrons reach the surface with an energy of only several electron volts $E_E = e(V_E + \Delta\varphi)$ where $\Delta\varphi$ is the difference in workfunction between the electron gun and the sample. The sample potential at which $E_E = 0$ is determined experimentally by the transition from mirror mode, where the electrons are reflected in front of the sample, to LEEM mode, where the electrons reflect from the sample proper.

As the sample is lifted to ≈ -15 kV, the power supply that is used to apply the in-plane bias voltage V_{bias} over the device has to be raised to the same potential. We use a home-built power supply that is integrated in the high-voltage electronics of the LEEM instrument. It allows us to vary V_{bias} from -10 V to 10 V at a maximal current of 100 mA.

The applied lateral field causes a slight tilt in the incoming and reflected electron beams. As mentioned in the main text, this is the cause for small deviations in the shape of the IV-curves. To keep the reflected beam on the optical axis of the microscope, we adjust the tilt of the illuminating beam accordingly. These tilted beams cause some image drift while scanning through V_E . We therefore apply a drift correction algorithm before creating the pixel-wise IV-curves.

Shift Determination

In the main text, we describe how we create a potential map of the sample by comparing the IV-curve at every pixel with a reference IV-curve taken at zero bias. Here we describe the applied computer algorithm, which determines the local potential in a two-step process. First, we determine the shift of the local IV-curves with respect to a reference curve obtained at the biased sample. Second, the shift of this biased reference curve to a reference curve acquired at unbiased conditions is quantified by determining the positions

of the minima in both curves. The local potential *V(x,y)* is then calculated as the sum of these two shifts. By using this two-step process, we overcome a difficulty introduced by the applied bias. The bias voltage gives rise to a lateral electric field, which the low-energy electrons cross on their way to the sample. Hence, their angle of incidence on the sample surface changes, which slightly alters the shape of the IV-curve. However, the reference IV-curves that are used in the first step of the process are altered in a similar fashion. Therefore, our two-step process is robust against the field-induced change of the IV-curves.

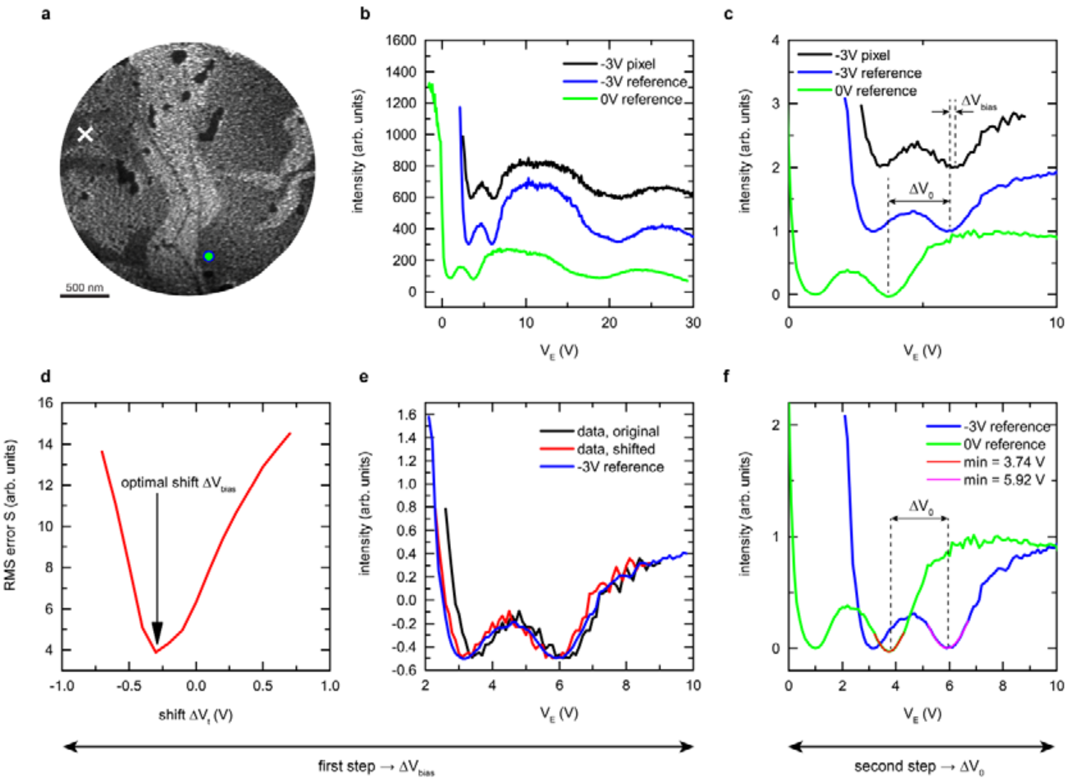

Supplementary Figure 1 | The local potential is found by calculating the shift of local IV-curves with respect to a reference in a two-step process. a, LEEM image showing the positions where the IV-curves where taken. **b**, Raw data of a local IV-curve (black) from a single pixel (white cross in **a**) on the biased sample, a reference curve taken at a bias of $V_E = -3$ V (blue) and on the unbiased sample (green). For the latter two, 2500 pixels are averaged in the area indicated by circles in **a**. **c**, The IV-curves in **b** are normalized, and cropped for the following shifting procedure. **d**, For various shifts of the local IV-curve with respect to the biased reference curve, the root mean square error is calculated. The minimum indicates the optimal shift ΔV_{bias}. **e,** Biased reference curve (blue), original data (black) and the curve shifted by the optimum found from **d** are shown. **f**, Finally, the shift ΔV_0 of the biased reference curve (blue) with respect to the reference curve of the unbiased sample (green) is obtained by finding the position of the high-energy minimum. This is done by fitting a Lorentzian function to the minimum. The total shift is the sum $\Delta V = \Delta V_{bias} + \Delta V_0$ of the two shifts.

Supplementary Fig. 1 shows how this is done in detail. We first determine the shift of an IV-curve at pixel *(x,y)* of a sample under bias $I_{x,y}^{bias}(V_E)$ with respect to a reference curve $I_{ref}^{bias}(V_E)$ from the same biased measurement. This yields relative potential differences on the sample. In a second step the absolute potential values are obtained by comparing $I_{ref}^{bias}(V_E)$ to a reference curve $I_{ref}(V_E)$ acquired from the unbiased case. In Supplementary Fig. 1, colors for curves are used consistently as follows: One particular LEEM IV-curve $I_{x,y}^{bias}(V_E)$ measured at an in-plane bias of $V_{bias} = -3\text{V}$ in a single pixel (indicated by the white cross in Supplementary Fig. 1a) is shown in black. The biased reference curve $I_{ref}^{bias}(V_E)$ (also measured at $V_{bias} = -3$ V) is obtained by averaging over ~2500 pixels in the bilayer area marked by a circle in Supplementary Fig. 1a and is shown in blue. The reference curve obtained from the unbiased sample taken at the same spot is shown in green.

Supplementary Fig. 1b shows the original data of a single pixel IV-curve together with reference curves for the biased and unbiased case as acquired from the LEEM measurement. Only the results for positive landing energies E_E are shown as for very low values of V_E the lateral electric field dominates and leads to artifacts. This is not problematic as we do not take the mirror mode into account for the following analysis. The overall intensity is different for curves taken at different positions due to inhomogeneous sensitivity of the detector. To make the curves comparable, they are normalized by setting the bottom of the minimum at highest V_E to zero and the following maximum to 1. Next, the curves are cropped in order to use only the most characteristic part of the IV-curve in the following analysis. This is the part around the minima that arise at specific energies due to the resonance of incoming electrons and graphene interlayer states. The used algorithm searches for the minimum in the IV-curve at the highest voltage below $V_E = 10\text{V}$ and crops the curves from there to a defined window. Note that this window is different for different layer numbers, which corresponds to a different number of minima and hence, a different relevant voltage scale. The reference curves of different layer numbers are also cropped but with a larger window. The normalized and cropped curves are shown in Supplementary Fig. 1c.

After preparing the IV-curves, we determine the shift in two steps. In a first step, the shift ΔV_{bias} of the IV-curve under study with respect to the biased reference curve is calculated. This is done by shifting $I_{x,y}^{bias}(V_E)$ by different amounts ΔV_t and optimizing the overlap with the reference curve $I_{ref}^{bias}(V_E)$. Therefore, the sum of the squared residuals

$$S = \sqrt{\sum_i \left(I_{x,y,i}^{bias}(V_E) - I_{ref,i}^{bias}(V_E)\right)^2}$$

between all data points i of the measured IV-curve $I_{x,y}^{bias}(V_E)$ and the biased reference curve $I_{ref}^{bias}(V_E)$ is calculated. The best shift ΔV_{bias} is found where this figure of merit is minimal. The evolution of S for different shifts ΔV_t is shown in Supplementary Fig. 1d. The minimum, indicating the best fit, is apparent. Supplementary Fig. 1e shows the biased reference curve (blue) together with the data before (black) and after shifting (red) by this optimal value ΔV_{bias}. The overlap of data curve and reference curve has obviously

improved. Calculating this best shift ΔV_{bias} for all points on the sample is equivalent to a map of the relative voltage drop within the sample under bias.

In a second step, the difference in electrical potential with respect to a ground electrode is determined. Therefore, the shift of the biased reference curve $I_{ref}^{bias}(V_E)$ with respect to a reference curve $I_{ref}(V_E)$ from the unbiased case is calculated. This is done by fitting a Lorentzian function to the minima at highest V_E for both curves. The difference between their centers is the second shift ΔV_0. This procedure is shown in Supplementary Fig. 1f. The total shift, which is the local potential at each point, is then obtained as the sum $\Delta V = \Delta V_{bias} + \Delta V_0$ of the two shifts determined in the two steps.
We perform this routine pixelwise separately for monolayer, bilayer and triple layer areas, e.g., we compare monolayer pixels to a monolayer reference curve, etc.

Supplementary Note

Advantages of using IV-minima instead of the mirror mode transition

It is not directly obvious why we chose the energetic position of the resonance-induced minima in the IV-curves as a *criterion* to determine the shift. As stated in the main text, any recognizable feature in the IV-curve would work. Therefore, the mirror mode transition (MMT) with its steep drop in intensity would be a natural choice. However, it turns out that features at higher energies give more reliable values for the local potential.

In Supplementary Fig. 2e we demonstrate that the energy resolution using the MMT is much lower than when using the resonances. This has several reasons. First, the MMT is much more sensitive to dirt particles (like resist residues), insulating areas (like buffer layer) and 3D structures (like step edges). This is a real-space effect as can be seen at the spots marked with arrows in Supplementary Fig. 2a,b (compare a and b). Therefore, taking the MMT as a criterion for LEEP reduces the lateral resolution of the technique. Related, the exact shape of the MMT in IV-curves depends on the local surrounding, for example the presence of imperfections in the vicinity (see Supplementary Fig. 2c,d). Therefore, the determination of the precise energy of the MMT becomes much harder. Finally, by taking higher-energy features instead of the MMT as a criterion, one can take more points of the IV-curve in consideration, thus reducing noise and enhancing the reliability of the criterion.

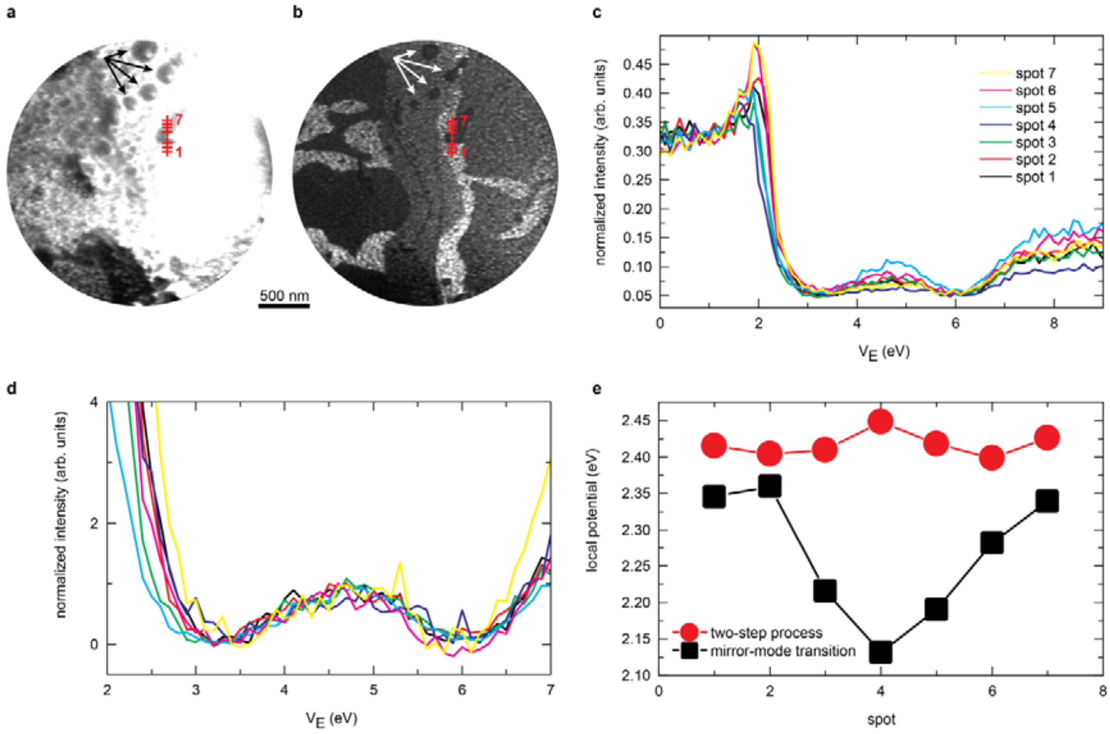

Supplementary Figure 2 | Advantages of resonant IV-curves. a, LEEM images close to mirror mode transition ($V_E = 2.4$ V) and **b** at somewhat higher energy ($V_E = 4.1$ V). The apparent size of dirt and other imperfections (see for example the spots marked with arrows) increases close to the mirror mode transition. **c**, IV-curves taken at the points marked in red in **a**,**b**. All have similar potential around a buffer layer area. The curves exhibit a clear difference in the exact shape of the drop of the mirror mode transition. **d**, The normalized IV-curves (procedure as described in Supplementary Methods: Shift Determination) coincide for higher energies, while the shift in mirror mode is even enhanced. **e**, The local potential, determined by fitting an error function to the data of the mirror mode transition in **c** (black squares), shows strong variations with position although the measurement spots lie on a equipotential line. The potential acquired by comparing the curve shape at higher energies (the method used in the manuscript) much less variation (red circles). This shows that taking higher energy features of IV-curves, like the minima due to resonant coupling, yields more robust results.

Supplementary Video 1

Drift-corrected bright-field LEEM movie of the sample with no in-plane bias applied, $V_{bias} = 0$ while changing the overall sample potential V_E (shown in the top-left). Upon changing V_E intensity changes can be observed that correspond to energy-dependent variations of the reflectivity. Bright corresponds to maxima, dark to minima in the LEEM IV-curves. Hence, for monolayer, bilayer and triple layer areas (layer number is indicated by numbers), we find one, two and three voltages below 10 V for which they become dark, respectively. The particular difference to Supplementary Video 2 is that all areas of the same layer number change color at the same V_E. Field of view is 2.6 μm.

Supplementary Video 2

Drift-corrected bright-field LEEM movie of the same area as in Supplementary Video 1, but now with an in-plane bias of $V_{bias} = -3$ V applied. The overall sample potential V_E (shown in the top-left) is varied from -1 V to 30 V. In contrast to Supplementary Video 1, the voltage V_E where intensity changes occur depends on the position now. The resulting contrast waves from left to right are a direct consequence of the in-plane voltage drop (most prominent from 3 V to 7 V). The vertical (in the movie) wave front is a representation of the equipotential lines in the sample. Field of view is 2.6 μm.
